# Prevalence and Associated Factors of Stunting among School Age Children in Addis Ababa City, Ethiopia 2021

**DOI:** 10.5334/aogh.3751

**Published:** 2022-07-21

**Authors:** Sindew Mahmud Ahmed, Sisay Shine, Genet Asefa, Melaku Belay

**Affiliations:** 1 Kotebemetropolitan University College of medical and health science nursing department, ET; 2 Deberbirhan University institute of medical and health sciences Public health Department, ET; 3 Kotebemetropolitan University College of medical and health science public health department, ET; 4 Kotebemetropolitan University College of medical and health science Surgery department, ET

**Keywords:** Stunting, Anthropometry, Schoolchildren, Addis Ababa, Ethiopia

## Abstract

**Introduction:**

Stunting has long been regarded as one of the most important indicators of malnutrition, serving as a proxy for not just chronic nutritional deficiency but also long-term socioeconomic disadvantage among children and society as a whole. In 2016, stunting alone afflicted an estimated 154.8 million (22.9%) children under the age of five over the world. It is one of Ethiopia’s most serious undernutrition and health problems among school-aged children.

**Objective:**

To determine the prevalence and associated factors of stunting among school-aged children in Addis Ababa city, Ethiopia 2021.

**Methods:**

An institutional-based cross-sectional study was conducted among primary school students in Addis Ababa city. By using a single population proportion, a formula of 627 students was recruited. From 11 sub-cities, 4 sub-cities were selected by lottery method, and 21 (30%) of the schools from the sub-city were selected. Finally, from each school, study participants were selected by using systematic random sampling, using their attendance list as a frame. A self-administered questionnaire was used to collect data, and anthropometric measurements were taken. In order to see the association between the dependent (stunting) and independent variables, bi-variable and multi-variable logistic regression were used. During bi-variable analysis, variables that had p-values of less than 0.2 were entered into multivariable analysis to see the effect of confounding factors. Adjusted Odds Ratios with 95% confidence intervals and a P-value of less than 0.05 were used to see the level of significance.

**Result:**

The prevalence of stunting was 108/607 (18.0%) with a 95% CI of 14.5–20.9). Being a male child (AOR = 0.616, 95% CI, 0.34–0.96), type of water source (AOR = 3.4, 95% CI, 1.12–10.37), not feeding breast milk (AOR = 3.411, 95% CI, 1.09–10.07), educational status, and ability to read and write (AOR = 2.11, 95% CI, 1.15–3.88) were predictors of stunting.

**Conclusion:**

The study showed that the prevalence of stunting was high, and it explored that stunting remains a noticeable attribute of urban school-age children. The higher educational status of the mother, exclusive breast feeding, using ground water, and being a female child were negatively associated with the prevalence of stunting. The risk of stunting was higher among male than female school-aged children. Findings from the study suggest the need to strengthen the strategies that lead to Sustainable Development Goal 4 to ensure all girls and boys complete primary and secondary schooling by 2030.

## Introduction

Undernutrition is a major public health issue, and stunting is a well-established public health indicator of chronic malnutrition that accurately reflects prior nutritional history as well as current environmental and socioeconomic conditions [5,13]. It’s a marker for a variety of pathological conditions linked to higher morbidity and mortality, reduced physical growth potential, decreased neurodevelopmental and cognitive function, and an increased chance of chronic disease in adulthood [1]. Malnutrition has an impact on human performance and health because it impairs physical growth, cognitive development, and physical labor capability [2].

Every year, one million children die as a result of stunting around the world [3]. Long-term implications of stunting in infancy and early childhood include reduced cognition and school performance, stunted physical development, poor health, and loss of independence. In most underdeveloped nations, stunting is a major public health issue that raises the risk of sickness and death throughout infancy [5,6]. It is a significant barrier to a child’s survival and development of full learning potential. This is why nutrition initiatives aimed at increasing childhood nutrition and encouraging linear growth [7] are so important [8]. It also comes at a high price for Ethiopia’s economy and society [4,9]. According to the cost of hunger report, about 67% of Ethiopia’s adult population was stunted as a kid, increasing the risk of various health problems and lower cognitive capacity, with stunting accounting for 16% of all Ethiopian primary school repetitions [3]. In Ethiopia’s food surplus areas, poor childhood feeding is the main risk factor [9]. Stunting is thought to be caused by malnutrition and is utilized as a public health indication of malnutrition (i.e., insufficient food and nutrients intake) [11]. It is caused by a number of different circumstances. These elements are interconnected and have a hierarchical relationship. Poor diet and disease are the primary determinants, both of which are caused by a number of underlying factors, including household food security, maternity and child care practices, access to health services, and a healthy environment. The basic socioeconomic and political situations have an impact on these underlying elements [6,12,13,14,15].

Stunting has a devastating and far-reaching impact on individuals and nations, ranging from impaired cognition, low school performance, and limited physical development to an increased risk of degenerative diseases like diabetes, as well as a negative impact on the economy by lowering working capacity and productivity [16,17,18,19].

Undernutrition, including stunting, is definitely a substantial contributor to child death, disease, and disability [20]. Stunting has been linked to poor developmental outcomes in young children as well as poor academic performance in older children [21].

Stunting is associated with child age, family size, mother’s education, father’s occupation, child’s immunization status, pre-lacteal feeding, and family planning utilization [18,22]. It is also linked to household food insecurity, feeding frequency, low socioeconomic status, poor sanitation, maternal postnatal vitamin supplementation coverage, and healthcare seeking behaviors [12,31,32]. In the past, school-based health and nutrition programs were implemented to tackle nutritionally related problems among school-aged children, but they were fragmented and uncoordinated due to being implemented by different stakeholders, which led to inefficient and ineffective program delivery as well as poor results [23].

Though there is a decline in the magnitude of undernutrition, in Ethiopia, about 44% of children under five are stunted and 10% are wasted [24]. However, literature is limited in its understanding of the magnitude of stunting in school-aged children. As a late complication of under-five stunting is obesity, determining the magnitude of stunting has a significant role in the prevention of obesity. Therefore, the aim of this study is to assess the prevalence of stunting and associated factors among children attending primary school (7–14 years) in public primary schools in Addis Ababa, Ethiopia.

## Methods and Materials

### Study design

An institutional based cross-sectional study was conducted among primary school students in Addis Ababa city.

### Population

#### Source population

All school-aged children attending public primary schools in Addis Ababa city.

#### Study population

All school-aged children attending randomly selected public primary schools of Addis Ababa city.

#### Study units

Selected school-aged children fulfilling the inclusion criteria.

### Eligible criteria

#### Inclusion criteria

A child who was attending public primary school from grade one to eight and age seven to fourteen in the selected school in Addis Ababa.

#### Exclusion criteria

Child with gross physical deformity difficult to measure their height, and those who are below seven was excluded from this study.

### Sample size determination

Sample size was calculated using single population proportion formula with the consideration of proportion of stunting, p = 47%, 95% CI, 5% maximum tolerable error, n = 383. Adding 10% no response rate using a design effect of 1.5, the final sample size was 627.

### Data Collection Methods

Data were collected using structured, pretested Amharic version questionnaires administered by health extension workers. The questionnaire was in six parts and included demographic characteristics of the child and the caregiver, the maternal condition during birth of the child, the housing quality and quality of water supply of the caretakers, and the dietary history of the child and the caregiver.

Data were collected in two phases. In the first phase, a self-administered questionnaire was sent to the parents or caretaker through the child or by the person taking the child home after class, then the child’s anthropometric data (height) was taken by data collectors.

Caregivers who could neither write nor read were instructed to seek assistance from their partner or somebody in the house to help them fill out the questionnaire.

Height was measured using standardized and calibrated equipment. The child’s was standing with his/her back against the measuring surface, with feet together flat on the floor, arms at side, and knees and back straight. Head, heels, buttocks, and shoulder blades touched the measuring surface. The child was looking straight ahead, and the headboard was slid gently down, compressing the hair. The height was written to the nearest 0.1 centimeter. The procedure was repeated a second time. Comparison was made between the two measurements. Accordingly, if the difference between the two measurement readings was within 0.1 centimeter, the second measurement was recorded. Otherwise, the average of the two measurements was taken.

Anthropometric-related data was transferred to anthro plus software, then it was exported to SPSS version 26. The Z-score of indexes, Height-for-Age Z-score (HAZ), was calculated using the World Health Organization (WHO) Multicenter Growth Reference Standard. The child was classified as stunted if his/her z score was less than –2SD and not stunted if Z score was ≥ –2SD.

To assure data quality, high emphasis was given in preparing data collection instruments. Due attention was given to selecting height measuring instruments and preparing questionnaires. Before starting the actual survey, the questionnaire was pretested on 45 individuals from the schools, which were not included in the study. The collected data were reviewed and checked for completeness before data entry. Incomplete data were discarded. The data entry format template was produced and programmed.

### Variables

The main outcome variable was stunting (yes/no), reflecting failure to receive adequate nutrition over a long period, resulting in low height-for-age at < –2SD of median value of the National Center for Health Statistics (NCHS)/WHO international growth reference [35]. The independent variables were sociodemographic characteristics of the child (age, sex); sociodemographic characteristics of the caretaker/mother head of household; mother’s age, education, background, monthly income; mother’s occupation; environmental and hygiene related factors (type of house, type of water source, toilet availability); health service utilization (history of mother related to number of children, antenatal care (ANC) follow up, family planning (FP) use, place of delivery, immunization, deworming); nutrition-related characteristics (dietary history of children/caregiver, exclusive breastfeeding (EBF), time of complementary feeding start, duration of breastfeeding); and health care characteristics (infection).

Exclusive breastfeeding is giving no other food or drink, even water, except breast milk in the first six months of age.

Weaning feeding is when children make the transition from breast milk to another source of nourishment.

School-aged means a child who is 7 to 14 years old.

Pre-lacteal feeding is defined as administration of any substances other than breast milk to newborn babies during the first three days after birth.

### Data management and analysis

After each questionnaire was checked for completeness, it was coded and entered using Epi-data version 3.1 Software. Then it was exported to SPSS version 26 for analysis. Data was cleaned to see completeness, presence of missing values, and outliers. Descriptive statistics (mean, frequencies, tables, and graphs) were used to summarize and describe the data. Both bi-variable and multivariable logistic regression analysis were used to check the association between the independent variables and the outcome variable (stunting). To test how well the model explains the data, Hosmer-Lemeshow Goodness of fit test was used. Bivariable analysis P-value of less than 0.2 were entered into the multi-variable logistic regression model. Both crude odds ratio (COR) and adjusted odds ratio (AOR) with 95% confidence level were computed to see the strength of associations. Multivariable logistic regression variables with P-value of less than 0.05 were declared significantly associated with stunting.

## Results

### Sociodemographic characteristics of participating children

The questionnaire was completed by 607 participants, giving a response rate of 96.8%. Nearly half (53.5%) of the study participants were female, and 547 (90.1%) of the study participants were less than 14 years old. The study also found that 468 (77.1%) mothers gave birth in health institutions (Table 1).

**Table 1:** Sociodemographic characteristics of school-aged children of Addis Ababa city.


VARIABLE	RESPONSE	N	%

Sex of the child	Male	282	46.5

	Female	325	53.5

Age of the child	Less than 14 years	547	90.1

	Greater than 15 years	60	9.9

Birth weight	Less than 2500 mg	150	24.7

	2500–4000 mg	429	70.7

	Greater than 4000 mg	28	4.6

	Institution	468	77.1

Place of delivery	Home	139	22.9


*Note*: N = frequency, % = percentage.

### Maternal or caretaker characteristics

The study revealed that about 387 (63.8%) of the households were led by fathers. About 310 (51.1%) of the mothers were housewives and 223 (36.6%) could read and write. It also showed the household income of 358 (59%) respondents was less than 3000 EBR. The majority of the study participants (461 or 75%) had four to six live children in the household. It also revealed that 499 (82.2%) were below the age of 28 when giving birth (Table 2).

**Table 2:** Maternal or caretaker characteristics in Addis Ababa city.


VARIABLE	CATEGORY	FREQUENCY	PERCENTAGE

Head of household	Father	387	63.8

	Mother	220	36.2

Maternal occupation	Housewife	310	51.1

	Self-employed	115	18.9

	Government employed	117	19.3

	Day laborer	65	10.3

Maternal educational status	Can’t read and write	75	12.4

	Can read and write	223	36.6

	Primary	124	20.4

	Secondary	109	18

	College and above	76	12.5

Monthly income (EBR)	Less than 3000	358	59

	3001–6000	181	29.8

	6001–9000	33	5.4

	Greater than 9001	35	5.8

Number of children	1–3	461	75

	4–6	133	21.9

	More than 7	13	2.1

Maternal age when giving birth	Less than 28 years	499	82.2

	More than 28 years	108	17.8


*Note*: N = frequency, % = percentage.

### Health care practice and feeding-related factors

The study also revealed that about 350 (57.7%) of the study participants had used family planning methods before pregnancy. Almost two thirds of the children received deworming, and three fourths of the children took vitamin A supplementation. A total of 517 people (85.2%) were fully immunized. Nearly 80% of the mothers had ANC follow-up during pregnancy, and 415 (68.4%) of the mothers used extra food during pregnancy. The major extra foods taken by the mothers during pregnancy were fruit and vegetables (29%) (Table 3).

**Table 3:** Health care practice and feeding-related factors.


VARIABLE	RESPONSE	N	%

Child dewormed	Yes	367	60.5

	No	240	39.5

Child take vitamin A	Yes	461	75.9

	No	146	24.1

Child complete immunization	Yes	517	85.2

	No	90	14.8

Family planning used before pregnancy	Yes	257	42.3

	No	350	57.7

ANC follow-up before giving birth	Yes	479	78.9

	No	128	21.1

Extra food during pregnancy	Yes	415	68.4

	No	192	31.6

Type of extra food	Cereals	129	21.3

	Meat and meat product	85	14

	Fruit and vegetable	176	29

	Fat and sweet	7	1.2

	Milk and milk product	80	13.2


*Note*: N = frequency, % = percentage, ANC = antenatal care.

### Environmental and hygiene-related factors

About 94.4% of the study participants lived in a house with a steel roof, while 342 (56.3%) lived in a house with a mud wall and floors. About 236 (38.9%) participants lived in a house with one room. Nearly two thirds of the study participants reported the toilet they had was made from cement, and about 80% of the study participants got water from a pipe. About 67.7% of the students had access to and sufficient water for daily use, and 48.4% of the household traveled less than 15 minutes to fetch water (Table 4).

**Table 4:** Environmental and hygiene-related factors.


VARIABLE	RESPONSE	N	%

Type of roof	Steel	573	94.4

	Clay	34	5.6

Wall and floor	Mud	342	56.3

	Cement	265	43.7

Number of rooms in the house	One	236	38.9

	Two	169	27.8

	Three	106	17.5

	Four	96	15.8

Type of toilet	Cement	410	67.5

	Wood	71	11.7

	Other	126	20.6

Source of water	Ground water	49	8.1

	Pipe	485	79.9

	Packed bottle	61	10

	Spring	12	2

Access to and sufficient water for daily use	Yes	408	67.2

	No	199	32.8

Time to fetch water	Less than 15 min	294	48.4

	15–30 min	160	26.4

	Greater than 30 min	153	25.2


*Note*: N = frequency, % = percentage.

### Feeding habits

The study revealed that about 208 (34.3%) of the study participants were fed only breast milk for less than six months. In this study, about 267 (44%) mothers responded that they started complementary feeding at six months. The majority of the mothers started with milk and milk products (222, 36.6%). About 391 (64.4%) mothers fed their children one to three times a day, and about 220 (36.6%) mothers fed meat three to four times per week. The study revealed that 435 (71.5%) mothers were fasting before the birth of the child. About 239 (54.3%) mothers fasted only during the fasting season (Table 5).

**Table 5:** Feeding habit and comorbidity of related characteristics.


VARIABLE	RESPONSE	N	%

Length of time breastfeeding	Not at all	24	4

	6–24 month	197	32.5

	Less than 6 months	208	34.3

	Moer than 24 months	176	29.3

Complementary feeding starting time	Before six months	90	14.8

	At six months	267	44

	After six months	191	31.5

	At four months	59	9.7

Type of complementary feeding food	Cereals	218	35.9

	Meat and meat products	33	5.4

	Fruits and vegetables	129	21.3

	Fats and sweets	5	8

	Milk and milk products	222	36.6

Daily feeding frequency	1–3 times	391	64.4

	4–6 times	158	26

	More than six times	58	9.6

Frequency of feeding meat	Daily	39	6.4

	3–4 times per week	220	36.2

	Monthly	348	57.3

Frequency of feeding fats and sweets	Daily	98	16.1

	Monthly	283	46.6

	3–4 times per week	226	37.2

Fasting before childbirth	Yes	435	71.3

	No	172	28.3

Time of fasting	During fasting season	239	54.3

	Wednesday and Friday	201	45.7


*Note*: N = frequency, % = percentage.

### Comorbidity and feeding style

The study also found that 12.2% of the children involved in the study had a history of coughing prior to the two weeks of the study. In this study, 42 (6.9%) of the children had a history of diarrhea two weeks prior to the study. During childhood, nearly half of the mothers (323 or 53.2%) fed their children with a spoon.

### Prevalence of stunting

Those respondents whose height for age was below 2 standard deviations were considered stunted. Of the 607 respondents, 108 (17.8.0%, with a 95% CI of 14.6–20.9) were stunted, and 499 (82.2%) were not stunted.

### Factors associated with stunting

In order to see the association between the independent and dependent variables, both bivariable and multivariable logistic regression models were fitted. In the case of bivariable logistic regression, all independent variables were entered into the model separately in order to select the candidate variables to be entered into the final model. From all the variables entered into the model, 13 variables were selected for the final model with a p-value less than 0.2. Finally, 13 variables were entered into the multivariable logistic regression model. From those variables entered into the final model, only four variables maintained their significant association with the outcome variable with a p-value less than 0.05.

The study showed the likelihood of stunting in a male child is 38.4% lower than the likelihood of its counterpart [AOR = 0.616, 95% CI (0.34–0.96), p-value 0.032]. Children from households getting water from a pipe were 3.4 times more likely to be stunted compared with children whose houses accessed ground water [AOR = 3.4, 95% CI (1.12–1037), p-value 0.031]. Children who were not breastfed were 3.41 times more likely to be stunted compared with those breastfed more than 24 months [AOR = 3.41, 95% CI (1.09–10.07), p-value 0.036]. Children whose mothers could read and write were 2.11 times more likely to be stunted than those whose mothers had completed secondary school or higher [AOR = 2.11, 95% CI (1.15–38.88), p-value 0.016] (Table 6).

**Table 6:** Bivariable and multivariable logistic regression analysis to identify determinants of stunting among primary school age children in Addis Ababa public primary schools, Ethiopia, 2021 (n = 607).


VARIABLE	CATEGORY	NUTRITIONAL STATUS		COR(95% CI)	AOR(95% CI)

		STUNTED (%)	NOT STUNTED (%)		

Sex	Male	61 (21.6)	221 (78.4)	1.62 (1.07–2.48)	0.616 (0.34–0.96)*

	Female	47 (14.5)	278 (85.5)	1.0	1.0

Types of water source	Pipe	93 (19.2)	392 (80.8)	0.267 (0.05–1.4)	3.40 (1.12–10.37)*

	Packed bottle	8 (13.1)	53 (86.9)	0.72 (0.19–2.78)	3.15 (0.79–12.52)

	Spring	3 (25.0)	9 (75)	0.45 (0.10–2.03)	3.37 (0.59–19.33)

	Ground water	4 (8.2)	45 (91.8)	1.0	1.0

Duration of breast feeding	Not at all	6 (25.0)	18 (75)	0.60 (0.22–1.64)	3.41 (1.09–10.7)*

	<6 month	33 (16.8)	164 (83.2)	0.81 (0.301–2.20)	1.12 (0.62–2.03)

	6–24 month	44 (21.2)	164 (78.8)	0.49 (0.18–1.35)	1.36 (0.77–2.40)

	>24 months	25 (14.0)	153 (86)	1.0	1.0

Maternal education	Can’t read and write	16 (21)	59 (78.7)	2.118 (1.04–4.33)	1.69 (0.78–3.62)

	Can read and write	51 (22.9)	172 (77.1)	2.32 (1.33–4.02)	2.11 (1.15–3.88)*

	Primary	20 (16.1)	104 (83.9)	1.52 (0.78–2.90)	1.19 (0.59–2.39)

	Secondary +	21 (11.1)	164 (88.6)	1.0	1.0


* P-value < 0.05.

## Discussion

The study was intended to determine the magnitude of stunting and associated factors among school-aged children in public primary schools in Addis Ababa. The study showed that the prevalence of stunting was 108 (17.8.0%, with a 95% CI of 14.6–20.9). This finding is lower than the study conducted in India (38.4%) [25], Wukero tawon (49.2%) [26], Libo-Kemekem district (49.4%) [2], Libo-Kemekem district (25.5%) [27], Yiregalem (35%) [10], West Gojam Zone (43.2%) [28], and Gondar town, northwest Ethiopia (46.1%) [29]. The possible reason for this variation may be due to a difference in study methods and existing nutritional programs. In addition, socioeconomic differences between areas (rural vs. urban, for example) could explain the differences in the prevalence of undernutrition across Ethiopia. Moreover, there might be a difference in dietary patterns and dietary diversity as well. This result is almost similar when compared to the study conducted in Addis Ababa (19.6%) [3], but which is higher when compared to the study conducted in Eastern Ethiopia (8.9) [7] and Nepal (13%) [30]. This inconsistency might be due to the variability of risk factors in different geographic regions, plus socioeconomic status and dietary diversity.

The study also found that male children have higher proportion of stunting than their counter part. Males were stunted at a rate of 61.6% (AOR = 0.616, 95% CI (0.34–0.96), p-value 0.032). This is supported by the studies in Addis Ababa [3], Harar, and Wollayeta [31]. This could be explained by height gain in females, which results in an increased height for age compared with their male counterparts.

The study showed that breastfeeding practice was significantly associated with stunting. This is supported by the fact that breastfeeding reduces consumption of complementary foods without an equivalent increase in human milk intake, thereby diminishing total energy intake [32]. It is also supported by the WHO recommendation, which explains that breastfed children over 12 months of age are less likely to become ill with some infectious agent [28]. It is also supported by breastfeeding. Decreased diarrheal incidence and increased breastfeeding were associated with an increase in length gain when dietary intake was low and diarrheal morbidity was high, implying that the likelihood of being stunted is high [32].

The study revealed that the odds of stunting are higher among children whose mothers can read and write compared to those mothers who attend secondary school and above. This is supported by the studies conducted in different parts of the world [12,29,33,34,35]. This could be explained by the fact that women with a higher education, owing to their exposure to the outside world, are more aware of personal hygiene and of promotive and curative health care than uneducated or less-educated women. Education can also enable women to make independent decisions and to have greater access to household resources. This finding contradicts the finding from Gonder that revealed children of educated mothers were more likely to develop stunting than children whose mothers were housewives [9].

In this study, there was a significant association between the source of water and stunting. Children from households getting water from a pipe were 3.4 times more likely to be stunted compared with children whose houses accessed ground water [AOR = 3.4, 95% CI (1.12–1037), p-value 0.031]. This could be explained by the fact that pipe water is surface water, and the likelihood of contamination in surface water is high, so children might suffer from infection.

## Conclusion and Recommendations

### Conclusions

Based on the findings of the current study, it can be concluded that the rate of stunting was high among school children in the study area, confirming that stunting still remains a public health problem in Addis Ababa, even though it was lower than the finding in 2014, which was 19.6% [3]. Lower educational status of the mother, exclusive breastfeeding, using ground water, and being a male child were predicators of stunting.

### Recommendation

The study showed that using ground water is protective against stunting. This calls for researchers to conduct a clinical trial or other kind of analytical study. It also revealed that duration of breastfeeding is a predictor of stunting, so the government needs to reconsider maternity leave from four months to six months. Since there is significant association between maternal education and stunting the minister of education strengthens the strategies that lead to sustainable development goal 4 to ensure all girls and boys complete primary and secondary schooling by 2030.

## Data Accessibility Statement

All data are already included in the manuscript.

## Figures and Tables

**Table:** 

AOR	Adjusted Odds Ratio
CI	Confidence Interval
COR	Crude Odds Ratio
DHS	Demographic Health Survey
EBF	Exclusive breast-feeding
ECCE	Early Childhood Care and Education schools
FP	Family Planning
H.H	House Hold
Ht/Age	Height for age
MOE	Ministry of Education
NCHS	National Center for Health Statistics
SD	Standard Deviation
SAC	School Age Children
SPSS	Statistical Package for Social Sciences
UNICEF	United Nations International Children’s Emergency Fund
WHO	World Health Organization
